# Locally Advanced Inflammatory Myofibroblastic Tumor Treated With Targeted Therapy: A Case Report and Literature Review

**DOI:** 10.7759/cureus.27223

**Published:** 2022-07-25

**Authors:** Charis Durham, Matthew Clemons, Alwin Alias, Kartik Konduri

**Affiliations:** 1 Hematology and Medical Oncology, Baylor University Medical Center, part of Baylor Scott and White Health, Dallas, USA; 2 Radiology, Baylor University Medical Center, part of Baylor Scott and White Health, Dallas, USA; 3 Hematology and Oncology, Baylor Scott and White Medical Center, Temple, USA; 4 Hematology and Medical Oncology, Texas Oncology - Baylor Charles A. Sammons Cancer Center, Dallas, USA

**Keywords:** anaplastic lymphoma kinase (alk) tyrosine kinase inhibitor, adjuvant tyrosine kinase therapy, crizotinib, alk fusion, neoadjuvant tyrosine kinase inhibitor therapy, epithelioid inflammatory myofibroblastic tumor, pulmonary inflammatory myofibroblastic tumor

## Abstract

Inflammatory myofibroblastic tumors (IMTs) are known to be associated with anaplastic lymphoma kinase (ALK) gene rearrangements. Other molecular alterations such as ROS proto-oncogene 1, receptor tyrosine kinase (ROS1), neurotrophic tyrosine receptor kinase (NTRK), and platelet-derived growth factor receptor (PDGFR) have also been identified in IMTs. Although there are no randomized controlled clinical trials comparing chemotherapy, tyrosine kinase inhibitors (TKIs), or other systemic therapies, the literature demonstrates the use of ALK-targeted TKIs as an effective strategy for the treatment of locally advanced or metastatic ALK-rearranged IMTs. This case report describes a patient with an ALK-rearranged locally advanced pulmonary IMT who was treated with neoadjuvant-intent crizotinib. The patient had a very favorable response to therapy, and surgery was declined. It is difficult to determine the duration and sequencing of TKI use in these settings as there is little published data to guide decisions. This report also includes a comprehensive compilation of published IMT cases with molecular alterations treated with systemic therapy, which also highlighted the duration of therapies and clinical outcomes.

## Introduction

Inflammatory myofibroblastic tumors (IMT) of the lung describe pulmonary lesions associated with inflammatory cell infiltration. Many of these tumors have a benign course, but some become invasive. This has led to a discussion as to whether IMTs are driven primarily by an inflammatory process or a neoplastic process with a notable inflammatory response [1-4]. Due to the tumor's histologic complexity, IMTs have been described by a variety of names (i.e., inflammatory pseudotumor, plasma cell granuloma) and identified in various anatomic locations, including the abdomen, pelvis, and retroperitum [5,6]. The histopathological description of IMT is a tumor with myofibroblastic mesenchymal spindle cells with inflammatory infiltration of plasma cells [7,8]. IMTs that have malignant features have also been described as sarcomas arising from mesenchymal tissue and presenting with a low mitotic count.

The treatment approach can be quite varied due to disease heterogeneity. Surgical resection is the ideal treatment strategy when feasible. Anaplastic lymphoma kinase (ALK) tyrosine kinase inhibitors (TKIs) have shown favorable responses when used in ALK-rearranged advanced pulmonary IMTs. We present a case of a young adult patient who had a locally advanced IMT of the lung and underwent neoadjuvant intent crizotinib therapy followed by an excellent response.

## Case presentation

A 19-year-old previously healthy man developed a cough and exertional shortness of breath in September 2019. Additional symptoms included intermittent fevers and 35-pound weight loss over the course of a year. He was initially treated with multiple antibiotics in the primary care setting without improvement in his symptoms. A chest X-ray in October showed a left hilar mass, pneumothorax, pneumomediastinum, and subcutaneous emphysema. He was admitted to the hospital and received acute management for pneumothorax. He then underwent bronchoscopy with transbronchial biopsies. The endobronchial biopsy of the left hilar mass revealed an inflammatory myofibroblastic tumor with immunostains positive for low molecular weight cytokeratin, epithelial membrane antigen, desmin, and ALK. Ki-67 showed a moderate proliferative index of 10-15%. Thyroid transcription factor-1 (TTF-1) and SRY-box transcription factor 10 (SOX-10) were negative. Fluorescence in situ hybridization (FISH) was positive for the ALK (2p23) gene rearrangement, echinoderm microtubule-associated protein-like 4 (EML4)-ALK in 54/100 cells examined (Vysis DNA probes, Abbott Molecular Inc., Des Plaines, US).

**Figure 1:**
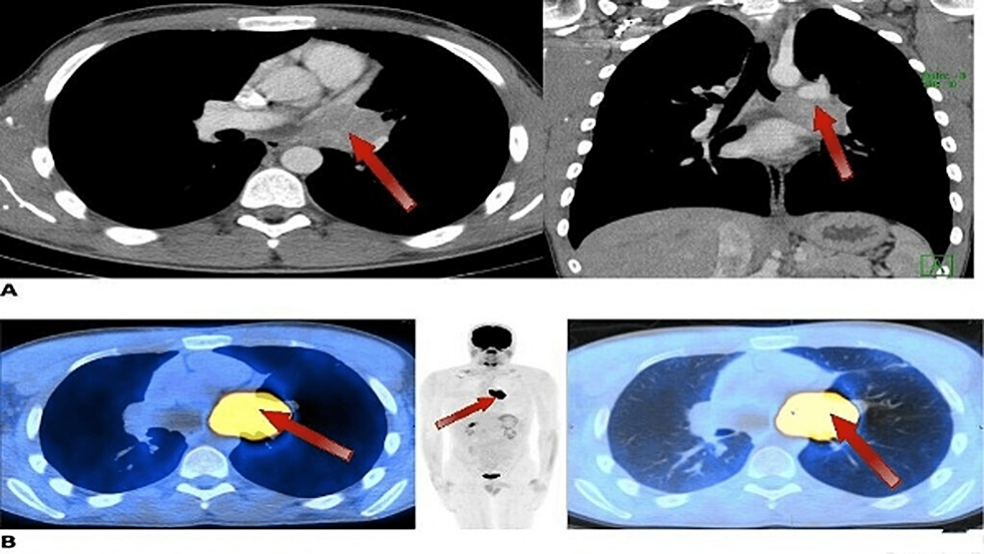
Pre-treatment imaging in January 2020 Axial and coronal CT images with contrast 1/21/20 (pane A) show a left hilar mass extending into the mediastinum measuring 4.3 cm x 3.1 cm that abuts the main and left pulmonary arteries, with an abrupt cutoff of the left upper and lower lobe central bronchi (indicated by red arrows). PET/CT fusion images (pane B) show the mass is intensely F-18 fluorodeoxyglucose (FDG)-avid (indicated by red arrows), with evidence of air trapping from the previously mentioned obstruction/cutoff of the bronchi.

After an initial delay in medical follow-up in the outpatient setting, a follow-up positron emission tomography (PET) scan in January 2020 visualized a left hilar mass measuring 4.3 cm x 3.1 cm, which was markedly hypermetabolic (Figure 1). There also appeared to be involvement of the left main pulmonary artery. The left mainstem bronchus was found to be occluded along with mucous plugging or mass extending into the left upper lobe and left lower lobe via dilated bronchioles. He was seen by thoracic surgery, and the patient's tumor was deemed borderline-resectable. He was started on steroids for symptom management and then referred to medical oncology for evaluation of a neoadjuvant approach. 

**Figure 2:**
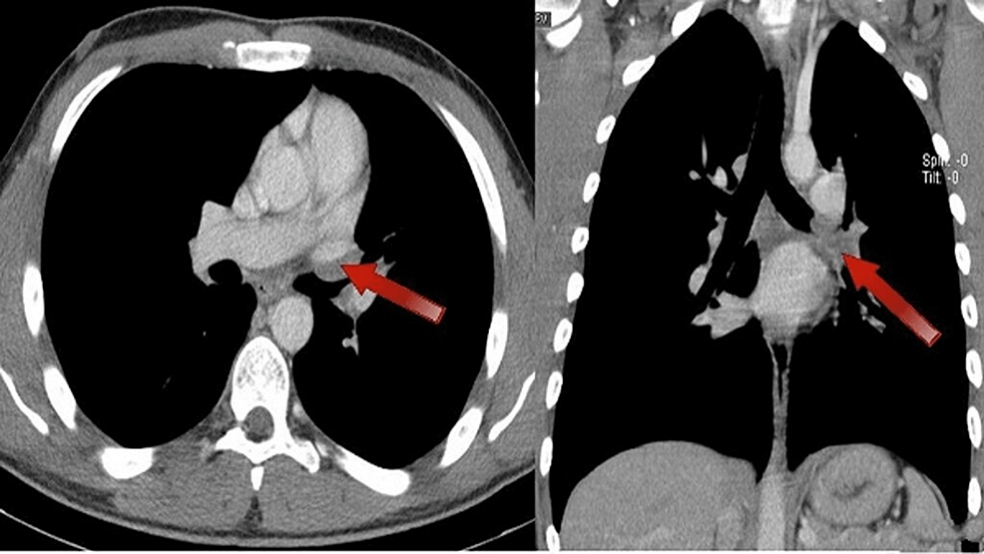
Axial and coronal images with contrast from March 27, 2020 (two months after initiation of therapy) Demonstrates a significant improvement in the mass, now measuring 2.5 cm x 0.9 cm (indicated by red arrows).

**Figure 3:**
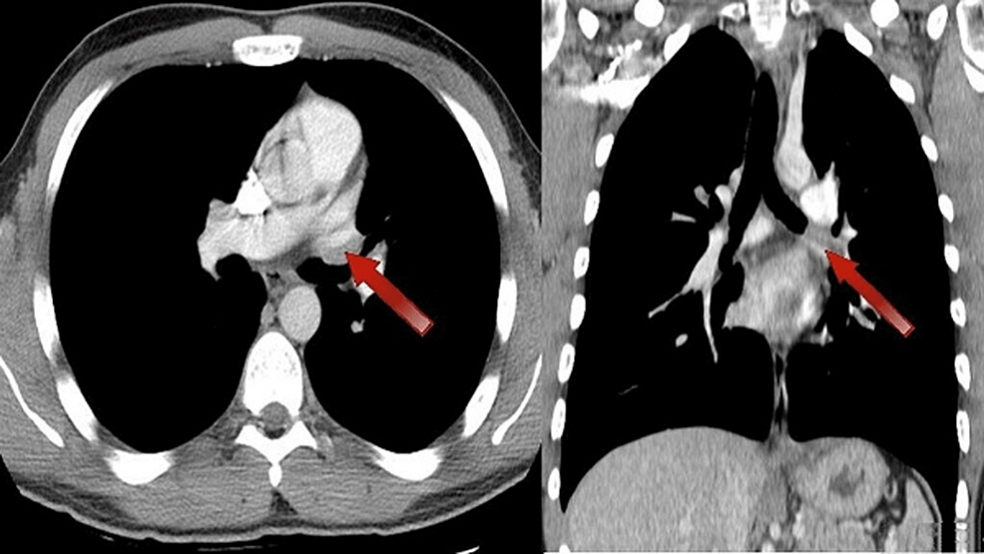
Axial and coronal CT images with contrast from June 26, 2020 (after four months of therapy) Further improvement in the mass, now measuring 2.0 cm x 0.5 cm (indicated by red arrows).

In the setting of an ALK gene rearrangement, the patient was weaned off steroids and started on crizotinib 250 mg twice daily. On initiation, he experienced mild nausea and an increase in serum creatinine level, which both resolved with antiemetics and increased fluid intake. Two months after initiating therapy, a CT scan with contrast was obtained (Figure 2). It showed a significant reduction in the size of the left hilar mass from 4.3 cm x 3.1 cm to 2.5 cm x 0.9 cm. Previously noted endobronchial filling defects were also resolved. A cardiac magnetic resonance imaging (MRI) was ordered by the thoracic surgeon, which demonstrated similar improvement in the hilar mass without left ventricular dysfunction or evidence of intracardiac involvement. A transthoracic echocardiogram was similarly unremarkable. A tumor board was held to discuss surgery versus continuing systemic therapy. Additionally, there was a patient-provider discussion, and the patient elected to continue systemic therapy in the setting of ongoing response. Follow-up imaging performed four months after therapy initiation showed further shrinkage in the area of the hilar mass to 2.0 cm x 0.5 cm (Figure 3).

The patient developed second-grade transaminitis and hepatic steatosis, which was evident on abdominal imaging. The patient had a history of alcohol usage but did report cessation while on therapy. Dose reduction of crizotinib was not required. The most recent CT scan from May 2022 demonstrated stable soft tissue thickening in the hilar area (2.0 cm x 0.5 cm), potentially representing treated versus residual disease. At the time this report was written, the tumor response lasted 28 months with ongoing crizotinib therapy.

## Discussion

IMT of the lung most often occurs in the first two decades of life. The diagnosis represents most of the pulmonary neoplasms in children under 16 years of age, but only around 1% or less of all bronchopulmonary tumors across all ages. Pulmonary IMTs have a variable presentation and prognosis. With surgical resection, patients can achieve an excellent disease-free response [9,10]. However, recurrences, even after prolonged remissions, have been noted [11]. Systemic therapies, including glucocorticoids, radiation, and chemotherapy, demonstrated mixed results in case studies [12,13]. Some cases have reported responses with non-steroidal anti-inflammatory drugs [14-16].

Approximately 36-60% of all patients with IMTs have an ALK gene rearrangement on chromosome 2p23 [17,18]. The presence of ALK rearrangements supports the notion that these IMTs are low-grade mesenchymal neoplasms with a secondary inflammatory component rather than merely a benign inflammatory process. Interestingly, one analysis suggested that ALK rearrangement may indicate a more favorable disease course as distant metastasis was primarily noted in IMTs lacking the ALK rearrangement [19,20]. TKIs that inhibit the ALK receptor, such as crizotinib, have been used in ALK-rearranged IMTs of the lung, abdomen, and pelvis with favorable responses [19-22].

Chemotherapy has also been studied, and one retrospective study reports an overall response rate of 50% in patients with locally advanced and metastatic disease [23]. A phase 2 non-randomized basket trial evaluating the efficacy of crizotinib in a variety of tumor types estimated a response rate of 66.7% in ALK-positive metastatic IMTs and a median progression-free survival (PFS) of 18.0 months (95% CI: 4.0-NE) [22]. There are no clinical trials comparing chemotherapy to TKI therapy in this setting. However, the toxicity profiles of ALK TKIs are generally more favorable.

Case reports also demonstrate the benefit of using TKIs against ROS proto-oncogene 1, receptor tyrosine kinase (ROS1) fusion-positive IMTs [24,25]. Neurotrophic tyrosine receptor kinase (NTRK) and platelet-derived growth factor receptor (PDGFR)-β fusions have been identified in IMTs, which further expands the potential for utilizing TKI therapy [26,27]. This highlights the importance of broad molecular testing, particularly in patients with ALK-negative IMTs, to further assess alternative molecular therapeutic targets. Table 1 summarizes a literature review on pediatric and adult cases of IMTs with molecular targets that were treated with TKI therapy [17-50]. Both metastatic and adjuvant/neoadjuvant cases were included. Only cases with clinical follow-up were included, and cases without documented follow-up results after TKI initiation were not included.

**Table 1:** IMT cases in the existing literature Cases of IMTs with identified molecular alterations were treated with systemic therapy with or without surgery. Cases without disclosure of clinical outcomes were excluded. If surgery was not described in cases of the advanced disease, the answer was presumed "no". The subtype of IMTs classified as epithelioid inflammatory myofibroblastic sarcoma (EIMS) was included if specified. Responses per Response Evaluation Criteria in Solid Tumors (RECIST) were not clarified in some reports. In these cases, categorization of response (i.e., partial response) was inferred based on the information given. M - male; F - female; CR - complete response; PR - partial response; SD - stable disease; PD - progressive disease; ALK - anaplastic lymphoma kinase; NGS - next generation sequencing; FISH - fluorescence in situ hybridization; IHC - immunohistochemistry; PCR - polymerase chain reaction; DOR - duration of response; chemo - chemotherapy; unk - unknown; yo - year-old; pt(s) - patient(s); EIMS - epithelioid inflammatory myofibroblastic tumor; NED - no evidence for disease; PFS - progression-free survival; NSAIDs - nonsteroidal anti-inflammatory drugs; ORR - overall response rate; CLTC - calthrin-heavy chain 1; CARS - cysteinyl-tRNA synthetase; TPM3 - tropomyosin 3; EML4 - echinoderm microtubule-associated protein-like 4

Study	Features	Location	Genotypic alteration	Testing modality	Therapy	Outcomes	DOR (months)	Surgery
Li et al. [17]	39 yo M, locally advanced disease	Pelvis	RANBP2-ALK	FISH, IHC	1: adjuvant chemo; 2: chemo embolization	1: disease recurrence after four months; 2: SD	2: 12	Yes
Kube et al. [19]	Nine pts, median age 9.1	Bladder, abdomen, head/neck, lung, extremity	ALK-fusion	IHC	1: chemo, NSAIDs, steroids, antibiotics; 2: crizotinib received by one pt in the second-line setting	1: one recurrence, two PD, three SD, two PR, one CR without surgery; 2: response (not defined) to crizotinib	1: One response followed by surgery and alive in CR1 at 7.6 years; 2: ongoing response to crizotinib at one year	Performed in pts with or without responses to frontline systemic therapy
Mosse et al. [20]	Seven pts; median age 10; advanced disease	Various	ALK fusion	IHC	Crizotinib	Three of six pts with measurable disease had PR	One pt with PR: 24	No
Passerini et al. [21]	Nine pts; median age 32; advanced disease	Unk	ALK fusion	FISH, PCR, or IHC	Crizotinib three pts had therapy prior to TKI	One CR, five PR, three SD	Two-year PFS 67% (29-138.3 weeks)	No
Schoffski et al. [22]	12 pts; median age 35.5; locally advanced and metastatic disease	Various	ALK fusion	FISH, IHC	Crizotinib some pts had prior systemic therapy	50% ORR	Median DOR: 9.0; duration of treatment: 7.2	No
Baldi et al. [23]	16 pts; advanced disease	Abdomen or lung	ALK fusion	IHC, FISH	Chemo	Eight of 16 patients evaluated had a response	PFS 4.7; overall survival of 22.4	No
Lovly et al. [24]	Eight yo M, advanced disease	Lung	TFG-ROS1 fusion	NGS	1: NSAIDs, steroids; 2: chemo; 3: crizotinib	2: unk 3: PR	3: four with ongoing response	No
Ambati et al. [25]	16 yo F, locally advanced; 10 yo F, locally advanced	Head and neck; lung	DCTN1-ALK; TFG-ROS1	NGS; NGS, PCR	Entrectinib 550mg/m2 daily; entrectinib	CR; PR	Ongoing response four months; ongoing response	Resection prior to TKI; no
Alassiri et al. [26]	17 yo F, locally advanced disease	Lung	ETV6-NTRK3	FISH, PCR, NGS	Multiple lines of chemo without response	PD after two cycles		Initial surgery followed by recurrence
Rafee et al. 2015 [28]	55 yo, locally advanced EIMS	Pelvis	ALK fusion	FISH	1: chemo; 2: crizotinib	1: NR; 2: PR	2: eight	Yes, crizotinib resumed adjuvantly
Nagumo et al. [29]	17 yo M, locally advanced	Bladder	ALK fusion	IHC, FISH	Neoadjuvant crizotinib	PR	Four then TKI stopped following surgery, no recurrence at one year	Yes
Gupta et al. [30]	32 yo M, advanced disease	Lung	ALK fusion	IHC	Neoadjuvant crizotinib	PR	No follow-up data	Presumably yes
Butrynski et al. [31]	44 yo M, advanced disease EIMS	Abdomen and pelvis	ALK-RANBP2	FISH and PCR	1: chemo; 2: crizotinib 200mg BID; 3: 250mg BID after second tumor debulking	1: PD; 2: PR; 3: achieved CR after tumor debulking	1: seven; 3: ongoing response at 30 months	Second tumor debulking for focal progression while on TKI
Trahair et al. [32]	Eight pts, median age 7, locally advanced and metastatic disease	Abdomen and pelvis	RANBP2-ALK SEC31A-ALK CLTC-ALK	IHC, FISH	1: perioperative crizotinib; 2: ceritinib for those with PD on crizotinib; 3: chemo	1: four CRs, three PRs, one SD; 2: one pt with CR on ceritinib for 3.5 years, one pt with PR on ceritinib then PD; 3: SD with eventual PD	Five patients: median duration of therapy of one year then stopped crizotinib without recurrence for average two more years	Yes
Debelenko et al. [33]	10 yo M, locally advanced	Chest	CARS-ALK	FISH, IHC	Neoadjuvant chemo and adjuvant radiation	PD		Yes and again after progressive disease
Saab et al. [34]	Six-month-old M	Abdomen	ALK fusion	FISH	Adjuvant chemo and radiation	Died of recurrent disease	36 months	Yes
Subbiah et al. [35]	Age in 50’s F, locally advanced disease	Pelvis	DCTN1-ALK	NGS	Crizotinib (250mg alternating days) and pazopanib (200mg daily) combination	PR	Six months ongoing response	Initial surgery before recurrence and TKI
Ono et al. [36]	57 yo M	Lung/pleura	RANBP2-ALK	IHC, FISH, PCR	1: ASP3026; 2: Ceritinib	1: PR; 2: PR	1: seven; 2: 11 then PD	No
Mansfield et al. [37]	32 yo M, metastatic disease	Multiple sites	TPM3-ALK	IHC, NGS	1: crizotinib; 2: ceritinib 750mg daily, dose reduced to 600mg due to toxicity	1: PR; 2: PR, followed by definitive therapy	1: eight; 2: 18, followed by eventual disease recurrence	After ceritinib underwent resection and ablation of sites of disease
Saiki et al. [38]	26 yo M, metastatic disease	Lung	EML4-ALK	FISH, IHC	1: chemo; 2: alectinib (600mg daily)	1: PD; 2: PR	2: four months with ongoing response	No
Yamamoto et al. [39]	22 yo M, locally advanced disease EIMS	Abdomen	RANBP2-ALK	IHC, PCR	Crizotinib	Alive with disease	10 months on TKI therapy	Initial surgery followed by recurrence and then TKI therapy
Lorenzi et al. [40]	24 yo M, locally advanced	Abdomen	CLTC-ALK	PCR, FISH	Crizotinib	SD	Four months with ongoing response	Initial debulking
Jacob et al. [41]	45 yo F, metastatic disease	Abdomen and spine	ALK fusion	FISH	Crizotinib	CR	27	No
Sarmiento et al. [42]	71 yo F, metastatic disease EIMS	Thorax	ALK fusion	FISH	1: crizotinib; 2: second-line ALK inhibitor	1: PR; 2: PR	1: two; 2: one year since surgery	Initial resection followed by progression and use of TKI
Liu et al. [43]	22 yo M Advanced disease EIMS	Abdomen	RANBP2-ALK	IHC, FISH	Adjuvant crizotinib	No recurrence after surgery and on TKI therapy	16 without recurrence	Yes
Yu et al. [44]	55 yo M; 22 yo M EIMS	Abdomen; abdomen	ALK fusion; ALK fusion	IHC FISH; IHC, FISH	Adjuvant chemo; crizotinib	Required repeat surgery and adjuvant chemo for recurrence PR	Free of disease at 10 months 14; alive with disease	Yes; initial surgery followed by recurrence and then TKI therapy
Ma et al. [45]	Seven yo M, EIMS	Abdomen	RANBP2-ALK	FISH	Neoadjuvant chemo followed by adjuvant chemo	Recurrent disease five weeks after chemo		Complete resection after neoadjuvant chemo
Gaudichon et al. [46]	16 yo F	Extremity	ALK positivity	IHC	NSAIDs, steroids, chemo, radiation, crizotinib	46 cumulative months of various therapy with mixed responses		Surgery after response to crizotinib
Theilen et al. [47]	Four yo F, locally advanced; 12 yo M, locally advanced	Liver; bladder	ALK positive; ALK positive	IHC; IHC	Crizotinib; crizotinib	CR; CR	Five, then crizotinib discontinued NED at 27; nine then crizotinib discontinued NED at 14	No
Shash et al. [48]	Nine months, locally advanced disease	Lung	TPM3-ALK	IHC, cytogenetics	Crizotinib, enoxaparin, ibuprofen	PR	Four months, patient then died from ARDS	Initial surgery
Kiratli et al. [49]	Seven yo F, locally advanced	Ocular	ALK positive	IHC	1: Crizotinib; 2: resumed crizotinib	1: CR; 2: second CR achieved	1: 12, then therapy stopped, recurrence three months after cessation; 2: 14 and ongoing	No
Reyes-Angel et al. [50]	Four yo M	Lung	ALK fusion	FISH	Adjuvant crizotinib (discontinued after one-year duration)	CR	Two years without disease recurrence (one year off TKI therapy)	Initial endobronchial resection and later ablation of residual tumor. These were prior to TKI use.

This case report exemplifies an attempt to use an ALK-directed TKI in a neoadjuvant approach for a pulmonary IMT with an ALK gene rearrangement. Therapy resulted in a near complete response and ongoing stable disease on surveillance imaging. In this case, surgery was declined. Of note, the optimal duration of targeted therapy in this context has not been defined.

There are a few published case reports investigating crizotinib in the neoadjuvant setting in adults. A patient with a large pelvic IMT and local involvement of the peritoneum but no distant metastasis was initially treated with chemotherapy without response [28]. The patient was then found to have an ALK gene rearrangement and was started on crizotinib 250 mg twice daily. Eight months later, there was a reduction in the size of the tumor (from 20 cm to 6.5 cm) on imaging. The patient then underwent surgery and restarted crizotinib therapy two weeks postoperatively with plans to continue indefinitely. No radiographically measurable disease was found at the six-month follow-up. Another case describes neoadjuvant crizotinib 250 mg twice daily used for a patient with IMT of the bladder, which resulted in a reduction in tumor size by 48% after two months [29]. The patient underwent partial cystectomy with negative surgical margins and no recurrence at the one-year follow-up. An abstract presents a patient with an ALK-rearranged pulmonary IMT who was treated with neoadjuvant crizotinib with the intention of surgery. The patient had a partial response [30]. Further data is not available on the results of this case. An adult patient with a metastatic ALK-rearranged abdominal IMT initiated crizotinib as second-line treatment and had a favorable response followed by progression in two localized areas. The patient underwent resection of these tumors and then reinitiated crizotinib. He had a complete response duration of 19 months with ongoing TKI therapy [31].

A review of pediatric literature describes patients (ages ranging from 7-14) treated with crizotinib in a locally advanced setting. Some of these patients had durable responses either with TKI therapy alone or with a TKI followed by surgery. In a small study, seven pediatric patients stopped the use of crizotinib after an average of one year of therapy (with or without surgery). Two of these patients had relapsed from their disease, while the others had a durable response at the time the study was published. The longest durable response while being off treatment was 3.7 years [32].

## Conclusions

Currently, targeted therapies are being studied in the neoadjuvant setting in pulmonary malignancies. Similarly, a neoadjuvant approach with ALK or other actionable genome-targeted treatments may have a role in pulmonary IMTs. This case suggests a unique approach in using targeted therapy in an adult patient with a borderline-resectable pulmonary IMT. In this context, further investigation is necessary regarding the comparison of systemic treatment options, sequence, and duration of therapy.
